# Mental Model Development in Multimedia Learning: Interrelated Effects of Emotions and Self-Monitoring

**DOI:** 10.3389/fpsyg.2019.00899

**Published:** 2019-04-24

**Authors:** Valentin Riemer, Claudia Schrader

**Affiliations:** Institute of Psychology and Education, Ulm University, Ulm, Germany

**Keywords:** interactive learning environments, serious games, mental models, metacognition, emotion

## Abstract

Learners’ emotions and metacognitive self-monitoring play a crucial role in mental model development, particularly in the context of multimedia learning. However, learning-centered emotions and self-monitoring have been investigated largely without accounting for their dynamic interrelations. In this study, the effects of both learner-state variables on mental model development were investigated, by modeling their interrelations over time during a multimedia learning episode. For this, 108 undergraduate students (*M*_age_ = 22.79, *SD*_age_ = 3.42) were engaged in a multimedia learning environment to learn practical money skills. Learning-centered emotions of enjoyment, boredom, and frustration were repeatedly collected using self-reports. Learners’ self-monitoring was assessed using behavioral data in terms of time spent on accessing specific information in the multimedia environment. Mental model development was operationalized by assessing learners’ mental model accuracy (MMA) in pre- and post-tests, by using assessments of structural knowledge. Regarding the dynamic interrelations, panel models with the repeated measures revealed positive direct and indirect paths from earlier stages of self-monitoring to later stages of enjoyment. Conversely, negative direct and indirect paths emerged from earlier stages of boredom and frustration to later stages of self-monitoring. Regarding the effects of all variables on mental model development, a path model analysis with aggregated values revealed that enjoyment was unrelated to post-test MMA, whereas boredom negatively predicted post-test MMA. Additionally, frustration negatively predicted self-monitoring, which positively predicted post-test MMA. Finally, pre-test MMA was a negative predictor of boredom and positively predicted post-test MMA. The results demonstrate that the dynamic interrelations between different learning-centered emotions and self-monitoring can diverge in multimedia learning. In addition, this study provides insights into the joint effects and the relative importance of emotions and self-monitoring for mental model development in multimedia learning.

## Introduction

Multimedia learning environments, defined as learning environments that provide content in verbal and non-verbal form (Mayer, 2005), enjoy lasting popularity. In particular, the advancement and accessibility of computer technology have put forth an increasing number of multimedia learning environments that respond to learners’ actions (Moreno and Mayer, 2007). Among the examples of such multimedia learning environments are serious games. Serious games are generally defined as computer games developed for purposes other than mere entertainment (Michael and Chen, 2006; Ritterfeld et al., 2009). They allow learners to actively engage with the learning content through animated elements that are under the learners’ control (Rieber, 2005). Additional examples of multimedia learning environments that learners can interact with are agent-based environments (e.g., Greene and Azevedo, 2009) or simulations (e.g., Darabi et al., 2009).

One of the major benefits of multimedia learning environments is seen in the promotion of meaningful learning, culminating in the acquisition of accurate *mental models* (Mayer, 2005; Moreno and Mayer, 2007; Sitzmann, 2011). Mental models can be defined as mental representations of how a knowledge domain is organized (van Merriënboer and Kirschner, 2017). They consist of knowledge contents, such as facts, concepts, plans or principles, as well as the structural relations between these contents (Kraiger et al., 1993; Jonassen, 1995; van Merriënboer, 1997). In the early stages of learning, learners’ mental models are usually inaccurate and based on intuition (Norman, 1983; van Merriënboer and Kirschner, 2017). To increase mental model accuracy (MMA), novel information must be integrated into existing models by establishing new structural relations and by dismissing previous misconceptions (Johnson-Laird, 1983; Norman, 1983; Seel, 2003).

The main function of mental models is seen as allowing learners to run internal simulations of processes in complex systems or tasks (e.g., technical or economic systems or mathematical tasks). Provided that these simulations are based on sufficiently accurate mental models, learners can correctly predict, evaluate and interpret the possible consequences of their actions (Johnson-Laird, 1983; Seel, 2003). Thereby, mental models facilitate the detection of problems that occur in complex tasks as well as the identification of pertinent solution strategies (Glaser, 1989; van Merriënboer et al., 1992; van Merriënboer and Kirschner, 2017).

Multimedia learning environments are assumed to facilitate mental model development by allowing learners to interact with complex systems (Wouters et al., 2009; Sitzmann, 2011; Koops and Hoevenaar, 2013). More specifically, such environments can foster learners’ active engagement with the learning material. The increased active engagement is argued to support learners’ cognitive processes necessary to integrate novel information into existing knowledge (Moreno and Mayer, 2007). Furthermore, multimedia learning environments can provide contextualized feedback on learners’ actions. Thereby, they can trigger cognitive conflicts, which foster the correction of misconceptions in existing mental models (Koops and Hoevenaar, 2013). For example, in a recent study by Kao et al. (2017), learners played a serious game to acquire principles of basic machinery physics. In this game, learners had to move a ball over a canyon by drawing and manipulating virtual objects, such as a catapult. The learners received feedback on their actions, which was contextualized in the game environment. For instance, a learner may have drawn a catapult with too short a lever, resulting in the ball falling into the canyon. Thus, a cognitive conflict may have been triggered concerning the observed consequence that contradicts the learner’s existing mental model.

The facilitation of mental model development through multimedia learning environments can be partially observed in empirical work, such as the above-mentioned study by Kao et al. (2017). In fact, the authors reported that learners showed a significant increase in MMA, operationalized as the similarity between learners’ and experts’ concept maps, from pre- to post-testing. In another study by Eseryel et al. (2013), learners played a serious game to develop scientific problem-solving skills. The authors found a significant increase in learners’ MMA, assessed in pre- and post-tests by calculating the similarities between learners’ and experts’ annotated causal representations. In contrast to these results, other studies have found only partial changes in mental models resulting from multimedia learning (e.g., Darabi et al., 2009; van der Spek et al., 2011; Riemer and Schrader, 2016a). For example, in Riemer and Schrader (2016a) reported focal changes in learners’ mental models after playing a serious game about financial education. However, no significant increase in overall MMA (i.e., similarity between learners’ and experts’ knowledge structures) was found. In another study, Darabi et al. (2009) let learners interact with a simulation for chemical engineering training. The authors found that learners’ MMA (i.e., similarity between learners’ and experts’ IF-THEN statements) only increased after an introductory stage. However, no further increase in MMA emerged after subsequent stages, in which the learners actually interacted with the simulation (Darabi et al., 2009). Finally, van der Spek et al. (2011) had learners playing a serious game related to medical triage training. The authors reported an increase in MMA (i.e., a similarity between learners’ and experts’ knowledge structures) from pre- to post-testing only for novice learners. In contrast, advanced learners showed no significant changes in MMA (van der Spek et al., 2011).

In light of the inconclusive findings reported above, some authors have stressed the need to take into account additional learner-related factors, which are involved in multimedia learning (e.g., Darabi et al., 2009; Eseryel et al., 2013; Riemer and Schrader, 2016a). In particular, within the framework of the *Cognitive-Affective Theory of Learning with Media* (CATLM) (Moreno, 2005; Moreno and Mayer, 2007), Moreno posits that the effectiveness of multimedia learning environments depends, to a large part, on affective and metacognitive factors. In particular, learner-state variables, such as learning-centered emotions (e.g., Linnenbrink and Pintrich, 2004) and metacognitive self-monitoring (e.g., Greene and Azevedo, 2009), play an important role in mental model development as well. Finally, emotions and self-monitoring are also expected to be interrelated (Efklides, 2011; Pekrun and Linnenbrink-Garcia, 2012). Nevertheless, research in the context of multimedia learning has largely focused on separately investigating either emotions or self-monitoring (e.g., Moos and Azevedo, 2008; Greene and Azevedo, 2009; Baker et al., 2010; Sabourin et al., 2012; Shute et al., 2015; Riemer and Schrader, 2016a). Thus, little is known about how learning-centered emotions and self-monitoring jointly predict the development of accurate mental models. Likewise, there is a lack of understanding about the underlying dynamic relations between these two learner-state variables as they unfold over time during multimedia learning.

This study attends to this research gap by modeling how learning-centered emotions together with self-monitoring affect mental model development in multimedia learning. Accordingly, in the next sections, the separate roles of both learner-state variables are discussed, followed by describing their relations with each other. In particular, understanding the temporal dynamics between them constitutes a precondition to investigate the effects of both learner-state variables in combination.

### The Role of Learning-Centered Emotions in Mental Model Development

Emotions are largely defined as short, affective episodes that occur in response to a specific stimulus object or event (Rosenberg, 1998). They can be described in terms of several dimensions, such as valence (positive vs. negative) and arousal (activating vs. deactivating) (Russell and Barrett, 1999; Russell, 2003; Pekrun and Linnenbrink-Garcia, 2012). In the context of learning, Pekrun further differentiated specific learning-centered emotions in his *Control-Value Theory of Achievement Emotions* (Pekrun, 2000, 2006). According to Pekrun (2006), achievement emotions are directly tied to an achievement-related outcome or to an achievement-related activity. The emotions investigated in this study pertain to an achievement activity (i.e., a multimedia learning episode). Thus, the focus is on enjoyment, boredom and frustration, which are the most commonly investigated activity emotions (e.g., Pekrun, 2006; Pekrun et al., 2017; Putwain et al., 2017). Moreover, these emotions belong to the category of emotions that are known to be widely experienced in multimedia learning environments, such as serious games (e.g., Poels et al., 2007; D’Mello, 2013; Harley et al., 2013). Enjoyment, for example, is usually experienced when a challenge matches the learners’ skills and when predefined goals are being met (van Lankveld et al., 2010; D’Mello and Graesser, 2011). Conversely, boredom often arises when the learning material has low perceived value, when learners have little control over the learning task, or when the challenge is too low compared to the learners’ skills (van Lankveld et al., 2010; D’Mello, 2013). Finally, frustration can be triggered when learners become stuck or repeatedly fail to accomplish a goal because their skill or knowledge is too low compared to the challenge (Gilleade et al., 2005; Kapoor et al., 2007; van Lankveld et al., 2010). In addition, learners’ emotions during multimedia learning may be affected by their initial MMA prior to learning, by modulating the amount of perceived control over the learning episode (see Pekrun, 2006). Empirically, support for this notion comes from the contexts of classroom learning (e.g., Pekrun et al., 2014; Putwain et al., 2017) as well as multimedia learning (e.g., Shute et al., 2015).

With regard to the role of learning-centered emotions in mental model development, it is argued that positive emotions activate general mental models (Bless, 2000) and increase attentiveness to the task at hand (Pekrun et al., 2002, 2004). Thereby, positive emotions should facilitate the integration of novel information into existing models, which leads to increased MMA (Bless, 2000). However, this view is only partially supported by empirical results in the context of classroom learning. For example, Broughton et al. (2012) reported the beneficial effects of general positive emotions on acquiring knowledge on elementary conceptual astronomy after a reading task. In contrast, Linnenbrink and Pintrich (2004) reported no relation between general positive emotions and conceptual change after reading texts on topics from advanced physics. In the context of multimedia learning, even negative relations between positive emotions and mental model development have been reported, such as by Jackson and Graesser (2007). In their study, learners interacted with an intelligent tutoring system on conceptual physics. The authors found that the more learners enjoyed the learning episode, the worse their understanding of conceptual physics was in a post-test (Jackson and Graesser, 2007). Negative emotions, on the other hand, can cause a lack of attention and low intrinsic motivation as well as leading learners to focus more on situational details and engage in superficial information processing (e.g., Baker et al., 2010; Pekrun et al., 2010, 2011; Sabourin et al., 2012). Thus, negative emotions are generally assumed to prevent the activation of a holistic mental model and the integration of new information (Bless, 2000). However, discrete negative emotions may have diverging effects on mental model development. For example, some researchers (Kort et al., 2001; Pekrun et al., 2007, 2017; D’Mello and Graesser, 2014) argue that mild levels of frustration can lead to increased mental effort, elaboration and critical thinking. Thereby frustration may facilitate problem-solving, and increase MMA. Empirically, in the study by Broughton et al. (2012), a negative relation between general negative emotions and conceptual knowledge was found. Likewise, Linnenbrink and Pintrich (2004) found support for the detrimental effects of negative emotions, which were negatively related to conceptual change. Regarding the possible beneficial effects of negative emotions, partial support comes from Shute et al. (2015). The authors found that the frustration experienced by learners during a serious game about conceptual physics was positively related to task performance in the game. However, frustration was unrelated to an understanding of physics as assessed in a post-test (Shute et al., 2015).

The inconsistencies in the above-mentioned findings indicate that the role played by emotions in mental model development is far from understood. In particular, the role of negative emotions, such as frustration, appears to be ambivalent, given the seemingly contradicting results (cf. Linnenbrink and Pintrich, 2004; Broughton et al., 2012; Shute et al., 2015). This lack of understanding may originate from the complex interactions between learning-centered emotions and other learner-related traits or states (see Pekrun, 2006). Thus, considering additional learner-related variables, such as self-monitoring, as well as initial MMA as a controlling factor, can help to unravel the complex mechanisms constituting the role of learning-centered emotions.

### The Role of Self-Monitoring in Mental Model Development

Self-monitoring is generally defined as a metacognitive process that targets the flow of information about a learner’s own cognitions (Nelson, 1996). It encompasses processes of “identifying the task, checking and evaluating one’s progress, and predicting the outcome of that progress” (Schmidt and Ford, 2003, p. 407). According to the *Metacognitive Affective Model of Self-regulated Learning* (Efklides, 2011), self-monitoring takes a central role in online task processing, as it informs the activation of metacognitive control. More specifically, information coming from self-monitoring takes the form of subjective experiences, such as cognitive interruptions or conflicts. Subsequently, these experiences trigger control processes, such as the allocation of time and effort (Efklides et al., 1999; Efklides, 2011).

While self-monitoring is a key component for learning in general (Winne and Hadwin, 1998; Efklides, 2011), its function of enabling learners to identify and handle cognitive conflicts is of increased relevance for mental model development (Greene and Azevedo, 2009; Roscoe et al., 2013). Cognitive conflicts arise to a heightened degree when learners encounter novel phenomena in complex systems or tasks (Merenluoto and Lehtinen, 2004). Only when these conflicts are detected through self-monitoring can misconceptions be identified and novel information integrated coherently into learners’ existing mental models (van Merriënboer and Kirschner, 2017).

The importance of self-monitoring for mental model development becomes even more pronounced in the context of multimedia learning. Despite providing learners with feedback on their actions, multimedia learning environments can differ widely in the extent of guidance about how to process the provided information (Moreno and Mayer, 2007; Orvis et al., 2009). Although learners in such environments may also benefit from other metacognitive strategies, such as planning or evaluating the task outcome (Orvis et al., 2009), self-monitoring is of increased importance. This has been demonstrated empirically in studies investigating the influence of self-monitoring relative to other metacognitive strategies (e.g., Greene and Azevedo, 2009; Roscoe et al., 2013). For example, Greene and Azevedo (2009) investigated different aspects of self-regulated learning, such as planning, self-monitoring and strategy use during multimedia learning. Self-regulation aspects were operationalized as learners’ behaviors (e.g., learners assessing whether their set goals have been met) during learning with an agent-based environment about the circulatory system. The authors reported that, of all the aspects involved, self-monitoring behavior was the most important predictor of increased MMA (Greene and Azevedo, 2009). Using a similar learning environment, Roscoe et al. (2013) found that self-monitoring behavior (i.e., using a tool to formulate questions about the learning content) positively predicted MMA regarding climate change. Conversely, goal-setting behavior (i.e., using text-searching tools) was not related to changes in MMA (Roscoe et al., 2013). In the context of serious games, Riemer and Schrader (2016a) also used a behavioral assessment of self-monitoring (i.e., time spent in game phases that allowed monitoring of current progress). The authors fond that the amount of self-monitoring behavior positively predicted post-test MMA regarding practical money skills.

Although the above-mentioned findings point to the crucial role of self-monitoring, it is often applied insufficiently by learners during multimedia learning (see Greene and Azevedo, 2009; Roscoe et al., 2013; Riemer and Schrader, 2016a). Roscoe et al. (2013), for example, found that a majority of learners did not use design features that were specifically designed to facilitate self-monitoring (i.e., formulating questions about the content). Likewise, Riemer and Schrader (2016a) reported that learners used only a small proportion of their time during learning with the serious game on monitoring their progress. The determinants of learners’ engagement in self-monitoring, however, remain undiscovered to date. Besides design elements, such as scaffolds (see Roscoe et al., 2013), learner-state variables, such as learning-centered emotions, may influence self-monitoring during multimedia learning.

### Relations Between Learning-Centered Emotions and Self-Monitoring

The relation between learning-centered emotions and self-monitoring has been widely acknowledged in research about of self-regulated learning in the classroom context (e.g., Pekrun et al., 2002; Linnenbrink, 2007; Efklides, 2011; Pekrun and Linnenbrink-Garcia, 2012). The prevalent assumption is that emotions influence self-monitoring by modulating learners’ mode of cognitive processing (see Pekrun and Linnenbrink-Garcia, 2012). For example, positive emotions are known to foster cognitive flexibility (Isen, 2001), which is an important prerequisite for metacognitive processes (Pekrun and Linnenbrink-Garcia, 2012). In contrast, negative emotions tend to promote rigid and analytical thinking (Isen, 2001). Therefore, positive emotions are thought to be positively related to self-monitoring. In contrast, negative emotions are largely believed to have a negative relationship with self-monitoring (see Pekrun and Linnenbrink-Garcia, 2012).

Besides considering the influence of learning-centered emotions on self-monitoring, the effects of self-monitoring on emotions have also been discussed (e.g., Pekrun et al., 2002; Efklides, 2011). For example, Pekrun et al. (2002) suggested that engaging in self-regulation strategies, such as self-monitoring, may increase the experience of subjective control and, thus, induce positive emotions. In contrast, the experience of external control (e.g., by an instructor) may instigate negative emotions.

Research addressing the relations between leaning-centered emotions and self-monitoring is largely situated in the context of academic classroom learning (e.g., Perry et al., 2001; Pekrun et al., 2002, 2004, 2011; Mega et al., 2014). For example, Perry et al. (2001), found a negative correlation between learners’ self-reported boredom and self-monitoring during an introductory psychology course. Furthermore, Mega et al. (2014) showed that university students’ self-reported frequency of general positive emotions experienced while studying was positively related to metacognitive strategy use. In contrast, general negative emotions were negatively related to metacognitive strategy use, although this relation was weaker than in the case of positive emotions. Studies in the academic context demonstrate similar results (e.g., Pekrun et al., 2002, 2004, 2011). With regard to a single learning episode, Pekrun et al. (2017), for example, found a positive correlation between enjoyment and metacognitive strategy use during a reading task. In addition, a negative correlation was reported for boredom, whereas frustration was not significantly correlated with metacognitive strategy use (Pekrun et al., 2017).

While the above-mentioned studies seem to provide a clear picture regarding the relations between learning-centered emotions and self-monitoring, they largely neglect the temporal dynamics between these two learner-state variables. These temporal dynamics are of particular relevance in multimedia learning environments, in which the experience of learning-centered emotions can change rapidly in terms of intensity and persistence (D’Mello and Graesser, 2012). Moreover, some emotions, such as boredom, can persist over an elongated period during multimedia learning, whereas others, such as frustration, are more transient (Baker et al., 2010). However, large-scale surveys relying on single-time self-reports (Perry et al., 2001; Pekrun et al., 2002, 2004, 2011; Mega et al., 2014) or on aggregated scores (Pekrun et al., 2017) allow for only limited inferences about the relations over time. Therefore, variations in the relations between emotions and self-monitoring that occur according to the intensity or the persistence of an emotion (see D’Mello and Graesser, 2014) cannot yet be revealed.

### Present Study

The overarching aim of the present study was to reveal how learning-centered emotions and self-monitoring during multimedia learning predict mental model development. To this end, we asked learners to engage in a learning episode in the domain of financial literacy using a serious game. During the learning episode, learners repeatedly reported their experience of enjoyment, boredom and frustration as three learning-centered emotions of high relevance for multimedia learning. Self-monitoring was assessed over the course of the learning episode using a behavioral indicator. Finally, mental model development was operationalized as changes in MMA from pre- to post-test.

The relevance of emotions as well as of self-monitoring has been emphasized theoretically (Moreno, 2005; Moreno and Mayer, 2007) as well as confirmed empirically (e.g., Greene and Azevedo, 2009; Shute et al., 2015). However, the interrelations between these variables have been addressed only in the context of classroom learning and without considering their dynamics during an actual learning episode (e.g., Mega et al., 2014; Pekrun et al., 2017). In particular, research addressing the role of both variables simultaneously has been based mostly on the assumption of emotions influencing self-monitoring (e.g., Mega et al., 2014). Consequently, possible opposite effects of self-monitoring influencing emotions (see Pekrun et al., 2002; Efklides, 2011) have been ignored. Therefore, as a precondition for reaching our research aim, we first addressed the nature of the relations between learning-centered emotions and self-monitoring during the multimedia learning episode. For this purpose, we applied a panel design approach, investigating the paths between the multiple measures of emotions and intermediate self-monitoring over time. Accordingly, the first research question and corresponding hypothesis were formulated as follows:

**Research Question 1:** How are the three learning-centered emotions and self-monitoring related to each other and what are the temporal dynamics between them during the multimedia learning episode?

Based on the findings stemming from research in the context of classroom learning, the corresponding hypothesis is that self-monitoring is positively related to enjoyment and negatively related to boredom and frustration. In addition, given the lack of research regarding the temporal dynamics between learning-centered emotions and self-monitoring, the question as to whether enjoyment, boredom and frustration predict self-monitoring, or whether self-monitoring predicts emotions, is addressed.

The findings in relation to the first research question were expected to inform the modeling of learning-centered emotions and self-monitoring for their effects on mental model development. This was done at an aggregate data level, in order to identify the general effects with a parsimonious model. Accordingly, the second research question and corresponding hypothesis were formulated as follows:

**Research Question 2:** How do learning-centered emotions and self-monitoring jointly predict MMA after the multimedia learning episode?

Given the theoretical assumptions regarding the isolated effects of emotions and self-monitoring on mental model development, the corresponding hypothesis is that post-test MMA is positively predicted by enjoyment, and negatively predicted by boredom and frustration. In addition, post-test MMA is assumed to be positively predicted by self-monitoring. These effects are assumed to emerge with initial MMA (i.e., pre-test MMA) being controlled for. In addition, it was expected that the interrelations between emotions and self-monitoring found at the panel data level also emerge in the aggregated overall model.

## Materials and Methods

### Participants

The sample consisted of 108 undergraduate students (56 females and 52 males) from a German university. Their mean age was 22.79 years (*SD* = 3.42). The participants came from the study fields of psychology (38.5%), STEM (science, technology, engineering, and mathematics) (32.2%), economics (19.2%) and medicine (8.2%). Regarding their socioeconomic status, 63.9% reported a monthly income below 500 euros, 25.0% reported an income between 500 and 1,000 euros, and 11.1% reported an income of more than 1,000 euros per month. These proportions are regarded to be representative of the income for the population of German university students (see Statista, 2018). The participants were recruited via e-mail invitations, announcements made in classes and notes posted on campus. For compensation, participants could choose to receive either a course credit or a payment of 15 euros. The participants gave their written informed consent in accordance with the Declaration of Helsinki. The review board of the authors’ institution declared that no ethics committee approval was required for this study.

### Stimulus – The *Cure Runners* Game

The multimedia learning environment used in this study was a serious game called *Cure Runners* (Three Coins, 2013). The instructional aim of *Cure Runners* is to enhance students’ financial literacy by training them in practical money skills. Practical money skills comprise, for example, planning ahead, financial decision-making and monitoring one’s own finances (Fessler et al., 2007). The corresponding mental models include knowledge related to, among others, budgeting, saving, credit and debt (Aprea, 2012). The Organisation for Economic Co-operation and Development (OECD) has repeatedly identified deficiencies in practical money skills among the general population (e.g., Atkinson and Messy, 2012; OECD, 2017) as well as among undergraduate university students (Bongini et al., 2016). Consequently, repeated calls for interventions have been raised to advance the practical money skills of university students (e.g., Miller et al., 2014; Gerrans and Heaney, 2016).

**FIGURE 1:**
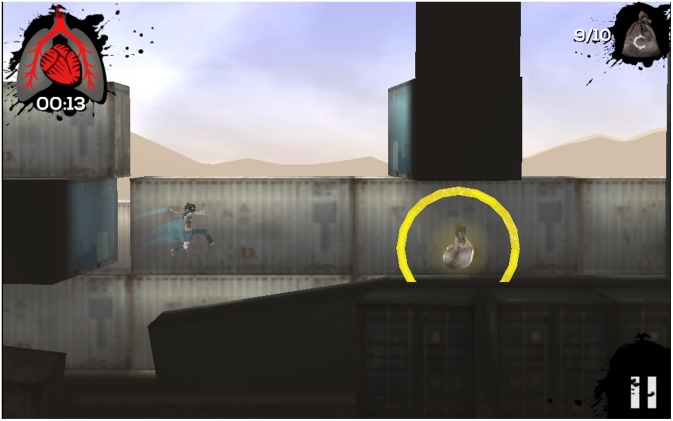
Example screenshot for a mission in *Cure Runners* (published with permission from Three Coins).

The platform game *Cure Runners* is set in a fictional world that suffers from an unknown infection. To survive in the world, learners need to collect *cure*, which serves as both a medicine and a currency in the game. The game comprises two major elements: (1) missions and (2) decision and reflection phases. To finish the game, learners must complete five consecutive sections. Each section comprises at least one mission and several decision and reflection phases. To progress from one section to another and to obtain cure, one mission must be accomplished in each section. The main objective of the missions (see Figure 1) is to guide the game character through the platform levels within a certain time. In addition, a certain number of specific mission items must be collected to complete a mission successfully. Within the platform levels, learners must overcome obstacles, such as jumping over abysses, to finish the mission in time. When time runs out, a small amount of cure is lost every additional second until the learner reaches the finish or chooses to cancel and possibly restart the mission. When learners run out of cure during a mission, the game character faints and the mission needs to be restarted.

**FIGURE 2:**
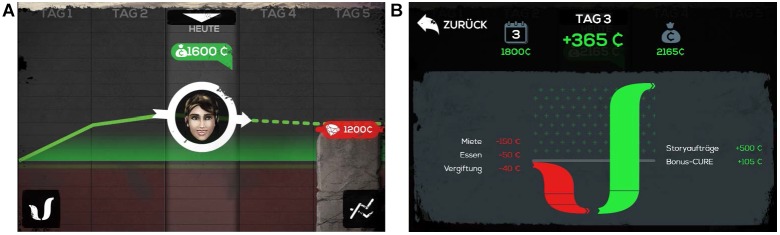
Example screenshots of **(A)** statistics screen with projection toward savings target and **(B)** balance sheet screen in *Cure Runners* (published with permission from Three Coins).

Between missions, learners can navigate through the decision and reflection phases. In these phases, learners must decide how to spend the cure by considering different types of expenses, such as regular expenses for housing and food, leisure expenses (e.g., alternative clothing for the game character) or expenses that arise from random events (e.g., injuries). In addition, learners can choose whether to pay immediately or postpone payment to later game sections (i.e., paying in installments or on credit), or not pay at all. Possible consequences of the learners’ decisions are becoming over indebted, not being able to pay for food and, as a result, becoming ill. In addition to minding their expenses, learners must meet a savings target (i.e., to pay a smuggler to leave the infested area) to finish the game successfully. Finally, learners can choose to view statistics and balance sheet screens (Figure 2) during the decision and reflection phases. These provide learners with information about their current amount of cure and a projection of their savings target (Figure 2A), as well as a comparison of earnings and spending (Figure 2B).

### Measures

#### Emotions

The three discrete emotions (i.e., enjoyment, boredom, and frustration) were assessed via self-reports using single items at multiple occasions during *Cure Runners*. Although the use of single-item measures of emotions has some disadvantages (see Harmon-Jones et al., 2016), they are less time-consuming and, thus, less prone to recall biases (Goetz et al., 2016). These benefits are of particular relevance in settings with repeated measures, as in the present study. Accordingly, single-item emotion self-reports have been used previously in research on multimedia learning with repeated measures (e.g., Sabourin et al., 2012), in experience-sampling studies (e.g., Goetz et al., 2016) and in cross-sectional designs (e.g., Goetz et al., 2006).

The items were administered on screen before the game as a baseline measure and after each of the five sections of *Cure Runners*. On each measurement occasion, participants had to respond to the statement, *I am currently experiencing…* followed by *enjoyment, boredom*, and *frustration* on three seven-point Likert scales ranging from 1 (*very little*) to 7 (*very strongly*).

#### Self-Monitoring

We used a behavioral measure to assess self-monitoring during learning. Previous studies have provided evidence for the predictive value of behavioral self-monitoring measures for learning outcomes in multimedia learning (Pieschl et al., 2012; Roscoe et al., 2013; Riemer and Schrader, 2016a). In particular, by following the definition of self-monitoring proposed by Schmidt and Ford (2003), we argue that the applied behavioral measure represents the amount by which learners checked and evaluated their progress on *Cure Runners*.

In this study, self-monitoring was operationalized as the ratio of time that learners spent viewing the statistics and balance sheet screens (see Figure 2) to the total time spent on navigating through the decision and reflection phases. This ratio was chosen over the absolute time spent viewing the statistics and balance sheet screens in order to account for missing values due to technical complications (see “Results” section). In addition, by comparing the time spent in the decision and reflection phases (instead of total playing time), the ratios remained unaffected by individual differences in gameplay proficiency regarding the missions.

The measurement of time was achieved using screen recordings of the individual learning sessions and a labeling software developed using *MATLAB* (The MathWorks, 2014). For each participant, the time spent on the statistics and balance sheet screens was divided by the time spent on the decision and reflection phases. The resulting ratios took the values of 0 < *x* < 1, with higher values indicating a higher amount of self-monitoring. This measure was also used in our previous study (Riemer and Schrader, 2016a,b) and correlated significantly with retrospective self-reports of self-monitoring (*r* = 0.23, *p* = 0.025, *n* = 97) as adapted from Schraw and Dennison (1994).

#### Mental Model Development

Mental model development was operationalized as changes in MMA, measured before (pre-test) and after (post-test) the learning episode. Pre- and post-test MMA was assessed using a structural knowledge assessment method based on the relatedness ratings of relevant domain concepts (e.g., Kraiger et al., 1995; Wouters et al., 2011). In the field of education, structural knowledge assessment has previously been used to assess mental models in a variety of topics (for an overview, see Trumpower and Vanapalli, 2016). Additionally, structural assessment has been applied in the context of multimedia learning to investigate mental model development in domains such as medical skills training (van der Spek et al., 2010, 2011; Wouters et al., 2011) and financial education (Riemer and Schrader, 2016a). This form of assessment is regarded as particularly suitable for representing the contextualized knowledge that multimedia learning environments, such as serious games, aim to promote (Wouters et al., 2011). Moreover, structural knowledge assessment has previously been shown to be a strong predictor of performance in transfer tasks (e.g., Kraiger et al., 1995; Day et al., 2001), thereby demonstrating its external validity.

**FIGURE 3:**
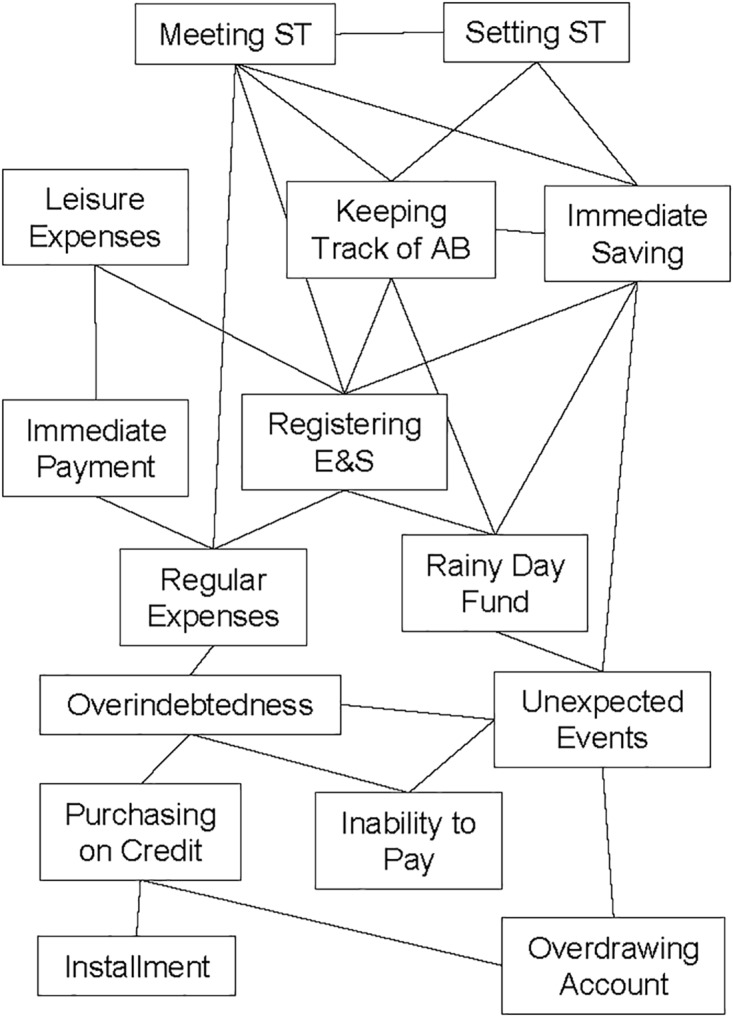
Referent network structure of practical money skills. ST, savings target; E&S, earnings and spending; AB, account balance.

In this study, the structural knowledge assessment was achieved in three steps. First, before and after the learning episode, the participants rated the relatedness of 105 pairs of domain concepts of practical money skills. The ratings were conducted via computerized questionnaires using seven-point scales, ranging from (1) *hardly related* to (7) *strongly related*. The 15 domain concepts (see Figure 3) were identified in collaboration with experts in financial education. Second, based on the relatedness ratings, network structures representing the individual mental models were established using the Pathfinder algorithm (Schvaneveldt(ed.), 1990). In these network structures, the domain concepts are represented as nodes and the relationship between the concepts as links (see Figure 3). The higher the relatedness ratings between concepts, the closer they are positioned to each other in the network structure (i.e., fewer concepts between them). The network structures were calculated using the *JPathfinder* software (Schvaneveldt, 2014). Third, all individual pre- and post-test network structures were evaluated for their accuracy (i.e., MMA) by comparison with a referent network structure of practical money skills (Figure 3). The referent structure was established based on the median scores of the relatedness ratings provided by three domain experts. We chose average expert ratings to establish a referent network structure because of the high validity provided by this approach (see Acton et al., 1994). MMA was computed as the degree of similarity between participants’ network structures and the referent structure using *JPathfinder* (Schvaneveldt, 2014). The resulting scores for MMA obtained values of 0 ≤*x* ≤ 1, with higher values indicating greater accuracy.

The MMA measure applied in this study has previously been shown to correlate with financial literacy measures adapted from Kempson (2009); Lusardi and Mitchell (2011), and the OECD (2012), largely in the expected directions (Riemer and Schrader, 2015). Significant positive correlations were found between MMA and self-reported savings behavior (*r* = 0.22, *p* = 0.031, *n* = 96) as well as problem awareness regarding debt (*r* = 0.31, *p* = 0.002, *n* = 96). In contrast, there was no significant correlation between MMA and numeracy skills (*r* = -0.16, *p* = 0.128, *n* = 96). However, this was not unexpected, since numeracy skills were not the focus of practical money skills as captured by the 15 domain concepts used for MMA assessment (see Figure 3).

### Procedure

The study was conducted in a computer laboratory on the university campus. The participants were informed that their learning sessions would be recorded and signed an informed consent form. An online questionnaire was subsequently administered comprising demographic information as well as the relatedness ratings from which the pre-test MMA was obtained. Participants then completed the first emotion questionnaire, which was used as a baseline measure of emotions. The participants then played *Cure Runners* and responded to the emotion questionnaires, which were administered during brief intermissions at the end of each of the five sections. At the end of the learning episode, the post-test MMA was obtained in the same way as the pre-test scores. The mean duration of the learning episode was 67.29 min (*SD* = 15.56) and the total procedure took approximately 90 min. Upon completion, participants were debriefed and received their compensation of choice.

### Analytical Approach and Statistical Analyses

**FIGURE 4:**
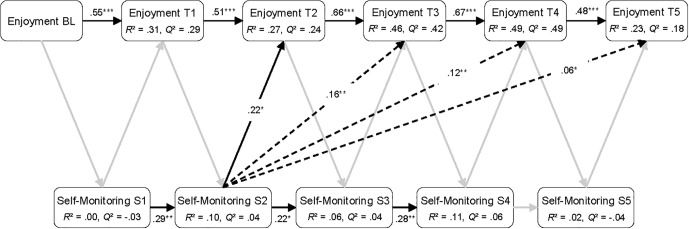
Sequential PLS-PM analysis showing all direct and the significant indirect paths between enjoyment and self-monitoring. BL, baseline measurement; T1 to T5, five measurement occasions during playing *Cure Runners*; S1 to S5, five sections of *Cure Runners*. Black lines, significant direct paths; gray lines, non-significant direct paths; dashed lines, significant indirect paths. *n* = 88. ^∗^*p* < 0.05, ^∗∗^*p* < 0.01, ^∗∗∗^*p* < 0.001.

**FIGURE 5:**
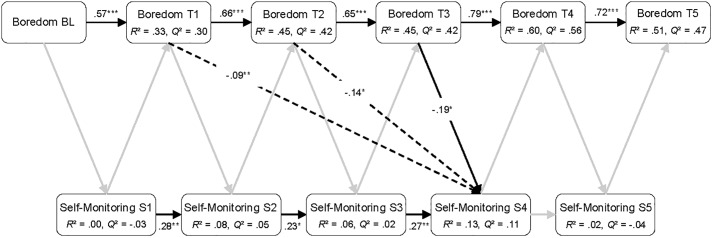
Sequential PLS-PM analysis showing all direct and the significant indirect paths between boredom and self-monitoring. BL, baseline measurement; T1 to T5, five measurement occasions during playing *Cure Runners*; S1 to S5, five sections of *Cure Runners*. Black lines, significant direct paths; gray lines, non-significant direct paths; dashed lines, significant indirect paths. *n* = 88. ^∗^*p* < 0.05, ^∗∗^*p* < 0.01, ^∗∗∗^*p* < 0.001.

**FIGURE 6:**
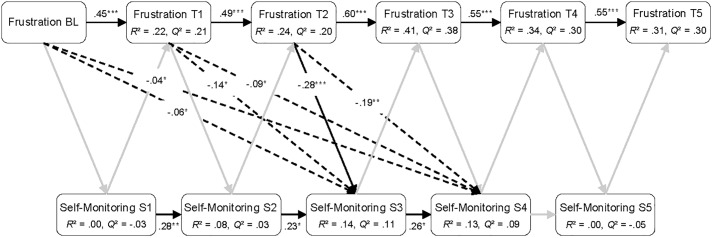
Sequential PLS-PM analysis showing all direct and the significant indirect paths between frustration and self-monitoring. BL, baseline measurement; T1 to T5, five measurement occasions during playing *Cure Runners*; S1 to S5, five sections of *Cure Runners*. Black lines, significant direct paths; gray lines, non-significant direct paths; dashed lines, significant indirect paths. *n* = 88. ^∗^*p* < 0.05, ^∗∗^*p* < 0.01, ^∗∗∗^*p* < 0.001.

The two research questions, as posed in Section “Present Study,” were addressed in two consecutive steps. In the first step, we used a panel design to address the first research question regarding the nature of the relations between the learner-state variables (i.e., emotions and self-monitoring) over the course of playing *Cure Runners*. Using sequential *Partial Least Squares Path Model* (PLS-PM) analyses (Wold, 1982, 1985), we established three separate panel models for enjoyment, boredom, and frustration, including the self-reported emotions measured at the baseline, as well as at the five measurement occasions during the game (T1–T5; see “Self-Monitoring” section). Each model also contained self-monitoring as measured during each of the five sections. It should be noted that, in contrast with classical cross-lagged panel models (e.g., Selig and Little, 2012), the variables were not assessed simultaneously on each measurement occasion, but in alternating order. Accordingly, paths were modeled for the self-reported emotions predicting self-monitoring in the subsequent section, for self-monitoring predicting the subsequent emotion self-reports, and between consecutive measures of emotions and self-monitoring (see Figure 4–6). This allowed for an investigation into the direct and indirect effects between variables, while controlling for autoregressive effects (Selig and Little, 2012). Similar panel design models have previously been used in conjunction with sequential path modeling techniques to investigate the directional influences between emotions and academic achievement (Pekrun et al., 2014; Putwain et al., 2017).

**FIGURE 7:**
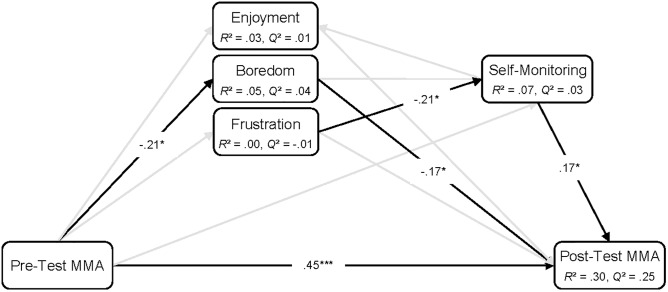
PLS-PM path model analysis showing paths between aggregated enjoyment, boredom, frustration, and self-monitoring, as well as pre-, and post-test MMA. MMA, mental model accuracy. Black lines, significant paths; gray lines, non-significant paths. *n* = 108. ^∗^*p* < 0.05, ^∗∗^*p* < 0.01, ^∗∗∗^*p* < 0.001.

In the second step, the second research question regarding the effects of learning-centered emotions and self-monitoring on mental model development was addressed. To this end, we applied a PLS-PM analysis, including values of the learner-state variables aggregated over the learning episode, and learners’ pre- and post-test MMA scores. For the emotions, we calculated the mean self-report scores over the five measurement occasions during the playing of *Cure Runners*. For self-monitoring, the ratios of the total time spent in statistics and balance sheet screens to the total time spent on the decision and reflection phases (see “Mental Model Development” section) were used. The aggregation of multiple measures is a common approach to capture the general effects between variables (see Bakdash and Marusich, 2017), which has been previously applied, for example, in the case of emotions (Spering et al., 2005; Shute et al., 2015) and engagement (Ben-Eliyahu et al., 2018). However, modeling the directional influences between aggregated variables is often based on assumptions, which are usually not tested (e.g., Shute et al., 2015). To counteract this weakness, we used the results from the first step regarding the temporal dynamics between emotions and self-monitoring to inform their positioning in the aggregate model (see Figure 7).

The application of PLS-PM analyses in both steps of the analytical approach has several advantages compared with covariance-based path modeling techniques. For example, the PLS-PM works efficiently with small sample sizes and complex models, makes no assumptions about the distribution of the underlying data and can handle single-item constructs without identification problems (Hair et al., 2013). Furthermore, being a variance-based approach, the PLS-PM is particularly useful for prediction-oriented research (Hair et al., 2013; Hamari et al., 2016), as is the case with the present study. However, in contrast with covariance-based approaches for model testing, there are no suitable criteria for global model evaluation (i.e., goodness of fit) available for the PLS-PM (Hair et al., 2013; Henseler and Sarstedt, 2013). Instead, the structural model was evaluated by inspecting the collinearity among the constructs, the significance of path coefficients (β) and the explained variance (*R*^2^), as suggested by Hair et al. (2013). In addition, Stone-Geisser’s *Q*^2^ values were examined, with values greater than 0, indicating a model’s predictive relevance for a given construct (Hair et al., 2013). Regarding effect sizes, we considered values of *R*^2^ larger than 0.02, 0.13, and 0.26 to represent small, moderate, and large effects, respectively (see Cohen, 1988). In addition, values of *Q*^2^ larger than 0.02, 0.15, and 0.35 can be considered to represent low, moderate, and high predictive relevance, respectively (see Garson, 2016). In the present study, no measurement models were needed to be evaluated, since we only used single-item constructs. The PLS-PM analyses were conducted using the *R*-packages *pls-pm* (Sanchez et al., 2017) and *semPLS* (Monecke and Leisch, 2013).

## Results

Due to technical complications, not all the measurement occasions of self-reported emotions or self-monitoring were recorded for some learners. As a result, the sample size varied between *n* = 88 for the panel models and *n* = 108 for analyses using the aggregated values.

### Descriptive Statistics and Preliminary Analyses

The means and standard deviations presented in Table 1 indicate that, from the baseline measures to about the midpoint of playing *Cure Runners*, the degrees of self-reported enjoyment and boredom appeared to decrease, whereas self-reported frustration appeared to increase. Repeated measure ANOVAs over the baseline measure and the five measurement occasions (T1–T5) revealed significant differences for enjoyment [*F*(5,83) = 6.62, *p* < 0.001, $\eta_{p}^{2}$ = 0.30], boredom [*F*(5,83) = 7.55, *p* < 0.001, $\eta_{p}^{2}$ = 0.31] and frustration [*F*(5,83) = 10.17, *p* < 0.001, $\eta_{p}^{2}$ = 0.38]. In subsequent pairwise comparisons, it was revealed that the degrees of self-reported emotions largely changed in the early stages of *Cure Runners* (see Table 1).

**Table 1:** Means and standard deviations for single measurement occasions of learner-state variables.

Variable	BL *M* (*SD*)	T1 *M* (*SD*)	T2 *M* (*SD*)	T3 *M* (*SD*)	T4 *M* (*SD*)	T5 *M* (*SD*)
Enjoyment	4.41^abcd^ (1.38)	4.09^abcd^ (1.33)	3.66^a^ (1.58)	3.42^b^ (1.67)	3.45^c^ (1.72)	3.39^d^ (1.90)
Boredom	2.70^abcd^ (1.46)	2.23^a^ (1.30)	1.95^a^ (1.22)	2.10^b^ (1.41)	2.15^c^ (1.41)	2.19^d^ (1.42)
Frustration	1.94^abcd^ (1.43)	2.40^abcd^ (1.51)	3.05^a^ (1.81)	3.33^b^ (1.91)	3.33^c^ (1.88)	3.00^d^ (1.89)
		
		**S1 *M* (*SD*)**	**S2 *M* (*SD*)**	**S3 *M* (*SD*)**	**S4 *M* (*SD*)**	**S5 *M* (*SD*)**
		
Self-monitoring		0.03^ab^ (0.01)	0.08^a^ (0.05)	0.07^b^ (0.06)	0.09^b^ (0.07)	0.04^ab^ (0.04)


*Measurement occasions with the same superscript are significantly different at *p* < 0.05. BL, baseline measurement. T1 to T5, five measurement occasions during playing *Cure Runners*. S1 to S5, five sections of *Cure Runners*. Range of scores: emotions: 1–7; self-monitoring: 0–1. *n* = 88.*

Self-monitoring appeared to be exhibited to a higher degree during the three middle sections compared to the first and last sections (see Table 1). There were significant differences in self-monitoring between sections [*F*(4,84) = 41.63, *p* < 0.001, $\eta_{p}^{2}$ = 0.67]. In addition, the amounts of self-monitoring differed significantly between almost all sections of *Cure Runners*, except between sections 2 and 3 as well as between sections 2 and 4 (see Table 1).

The means and standard deviations for the aggregated measures of self-reported emotions and self-monitoring, as well as the pre- and post-test scores for MMA, are shown in Table 2. In general, enjoyment, boredom, and frustration were reported to be experienced in low to moderate degrees throughout the course of playing *Cure Runners*. Furthermore, learners spent an average of 5% of their time during the decision and reflections phases on self-monitoring. Finally, learners’ MMA scores were generally moderate, given a hypothetical maximum of 1. However, MMA did not increase significantly as a result of playing *Cure Runners* [*t*(107) = -0.86, *p* = 0.391].

**Table 2:** Means and standard deviations for aggregated learner-state variables and pre- and post-test MMA.

Variable	*M* (*SD*)
Enjoyment	3.63 (1.28)
Boredom	2.16 (1.19)
Frustration	3.13 (1.42)
Self-monitoring	0.05 (0.03)
Pre-test MMA	0.29 (0.07)
Post-test MMA	0.30 (0.09)


*MMA, mental model accuracy. Range of scores: emotions: 1–7; self-monitoring: 0–1; MMA: 0–1. *n* = 108.*

### Interrelations Between Emotions and Self-Monitoring

Across all three panel models with the paths between single measurement occasions of emotions and self-monitoring, the variance inflation factors (VIFs) for the predictors ranged from 1.00 to 1.10 and the tolerance values ranged from 0.91 to 1.00. Thus, collinearity was not an issue for the sequential PLS-PM analyses (see Hair et al., 2013).

The results of the sequential PLS-PM analyses are reported in Figure 4–6. In the figures, the significant direct paths between the variables are shown as solid black lines, whereas the non-significant direct paths are shown as gray lines. The non-significant path coefficients are omitted from the figures for better readability, but can be found in Supplementary Tables S1–S3. In addition, the significant indirect paths (i.e., total compound effects of intermediate direct paths) are shown in the figures as dashed lines. However, the non-significant indirect paths are omitted for reasons of clarity.

#### Enjoyment

The bivariate Pearson correlations between the single measurement occasions of self-reported enjoyment and self-monitoring, shown in Table 3, were largely positive. Thus, learners who reported higher enjoyment while playing *Cure Runners* also engaged in more self-monitoring. However, only the correlation coefficients between enjoyment at T2 and T4 and self-monitoring in section 4 were statistically significant.

**Table 3:** Pearson correlations between single measurement occasions of enjoyment and self-monitoring.

Variable	1	2	3	4	5	6	7	8	9	10
1. Enjoyment BL	-									
2. Enjoyment T1	0.55^***^	-								
3. Enjoyment T2	0.29^**^	0.48^***^	-							
4. Enjoyment T3	0.33^**^	0.62^***^	0.68^***^	-						
5. Enjoyment T4	0.32^**^	0.48^***^	0.52^***^	0.69^***^	-					
6. Enjoyment T5	0.29^**^	0.48^***^	0.28^**^	0.55^***^	0.45^***^	-				
7. Self-monitoring S1	-0.03	0.06	0.06	0.01	-0.16	-0.10	-			
8. Self-monitoring S2	0.04	-0.14	0.15	0.02	-0.04	-0.08	0.28^**^	-		
9. Self-monitoring S3	-0.01	0.13	0.18	0.20	0.02	0.01	0.28^**^	0.24^*^	-	
10. Self-monitoring S4	0.05	0.16	0.22^*^	0.18	0.24^*^	-0.08	0.07	0.16	0.31^**^	-
11. Self-monitoring S5	0.01	-0.02	-0.01	0.06	0.15	-0.09	0.14	0.23^*^	0.17	0.03


*BL, baseline measurement. T1 to T5, five measurement occasions during playing *Cure Runners*. S1 to S5, five sections of *Cure Runners*. *n* = 88. *^∗^p* < 0.05*, ^∗∗^p* < 0.01*, ^∗∗∗^p* < 0.001.*

The results of the PLS-PM analysis are presented in Figure 4 (see also Table S1 in the Supplementary Materials). No significant paths from self-reported enjoyment in the direction of self-monitoring were found over the course of playing *Cure Runners*. In contrast, self-monitoring, as exhibited during section 2, was a significant positive predictor of enjoyment reported at T2 (i.e., after section 2). Additionally, there were significant positive indirect paths from self-monitoring to self-reported enjoyment. The indirect paths shown in Figure 4 represent the compound effects of all direct paths between self-monitoring in section 2 and enjoyment reported at T3, T4, and T5. Furthermore, enjoyment and, to a lesser extent, self-monitoring showed significant autoregressive effects. The *R*^2^ values presented in Figure 4 indicate that the variance explained by the predictors was moderate to high for self-reported enjoyment and small to moderate for self-monitoring. In addition, the *Q*^2^ values indicate that predictive relevance in the model was medium to high for all five measurement occasions of enjoyment and low for self-monitoring in sections 2 to 4. For self-monitoring in sections 1 and 5, predictive relevance was not given.

#### Boredom

The Pearson correlations between the measurement occasions of self-reported boredom and self-monitoring, shown in Table 4, indicate that boredom was generally negatively related to self-monitoring. Therefore, the more that learners reported being bored, the less they engaged in self-monitoring. However, only the correlation between boredom reported at T3 and self-monitoring shown during the subsequent section was significant.

**Table 4:** Pearson correlations between single measurement occasions of boredom and self-monitoring.

Variable	1	2	3	4	5	6	7	8	9	10
1. Boredom BL	-									
2. Boredom T1	0.56^***^	-								
3. Boredom T2	0.64^***^	0.66^***^	-							
4. Boredom T3	0.58^***^	0.48^***^	0.66^***^	-						
5. Boredom T4	0.47^***^	0.55^***^	0.64^***^	0.77^***^	-					
6. Boredom T5	0.52^***^	0.44^***^	0.62^***^	0.68^***^	0.71^***^	-				
7. Self-monitoring S1	0.06	-0.06	-0.08	-0.07^**^	-0.12	-0.07	-			
8. Self-monitoring S2	0.01	-0.03	-0.13	-0.02	0.09	0.02	0.28^**^	-		
9. Self-monitoring S3	-0.03	-0.01	-0.10	-0.18	-0.09	-0.00	0.28^**^	0.24^*^	-	
10. Self-monitoring S4	0.06	-0.03	-0.06	-0.24^*^	-0.08	-0.10	0.07	0.16	0.31^**^	-
11. Self-monitoring S5	0.05	0.08	0.03	0.14	0.13	0.05	0.14	0.23^*^	0.17	0.03


*BL, baseline measurement. T1 to T5, five measurement occasions during playing *Cure Runners*. S1 to S5, five sections of *Cure Runners*. *n* = 88. *^∗^p* < 0.05*, ^∗∗^p* < 0.01*, ^∗∗∗^p* < 0.001.*

The PLS-PM presented in Figure 5 (see also Table S2 in the Supplementary Materials) shows that boredom reported at T3 was a significant negative predictor of self-monitoring exhibited in the subsequent section (4). In addition, significant indirect paths were found for boredom reported at T1 and T2, negatively predicting self-monitoring in section 4. No significant paths of self-monitoring on the subsequent reports of boredom were found. Moreover, the autoregressive effects of boredom and self-monitoring were similar to those in the model with self-reported enjoyment and self-monitoring. The explained variance was high for self-reported boredom and low for self-monitoring, except for section 4 where the *R*^2^ value was of a moderate size. Predictive relevance was moderate to high for self-reported boredom and low for self-monitoring, except in sections 1 and 5, in which no predictive relevance was present.

#### Frustration

The Pearson correlations for the measurement occasions of self-reported frustration and self-monitoring are provided in Table 5, revealing largely negative correlations between frustration and self-monitoring. Thus, learners who reported more frustration also showed less self-monitoring. However, significant correlation coefficients were only found between the final four measurement occasions of frustration and self-monitoring during sections 3 and 4.

**Table 5:** Pearson correlations between single measurement occasions of frustration and self-monitoring.

Variable	1	2	3	4	5	6	7	8	9	10
1. Frustration BL	-									
2. Frustration T1	0.45^***^	-								
3. Frustration T2	0.23^*^	0.49^***^	-							
4. Frustration T3	0.31^**^	0.42^***^	0.63^***^	-						
5. Frustration T4	0.26^**^	0.42^***^	0.42^***^	0.58^***^	-					
6. Frustration T5	0.32^**^	0.41^***^	0.46^***^	0.56^***^	0.56^***^	-				
7. Self-monitoring S1	-0.02	-0.16	-0.06	-0.10	0.09	-0.03	-			
8. Self-monitoring S2	-0.12	-0.03	-0.03	0.02	0.11	-0.08	0.28^**^	-		
9. Self-monitoring S3	-0.07	-0.25^*^	-0.29^**^	-0.27^*^	-0.09	-0.17	0.28^**^	0.24^*^	-	
10. Self-monitoring S4	-0.01	-0.14	-0.18	-0.26^*^	-0.24^*^	-0.02	0.07	0.16	0.31^**^	-
11. Self-monitoring S5	0.03	0.05	0.10	0.07	0.04	0.08	0.14	0.23^*^	0.17	0.03


*BL, baseline measurement. T1 to T5, five measurement occasions during playing *Cure Runners*. S1 to S5, five sections of *Cure Runners*. *n* = 88. *^∗^p* < 0.05*, ^∗∗^p* < 0.01*, ^∗∗∗^p* < 0.001.*

The results of the PLS-PM analysis, presented in Figure 6, indicate that frustration reported at T2 was a significant negative predictor of self-monitoring during the subsequent section (see also Table S3 in the Supplementary Materials). Additionally, a series of significant indirect paths was detected. The dashed lines in Figure 6 represent the compound effects of all direct paths between frustration reported at the baseline, at T1 and at T2, in turn negatively predicting self-monitoring in sections 3 and 4. No significant paths were found for self-monitoring in the direction of self-reported frustration. The autoregressive effects in the model resemble those reported for the models with self-reported enjoyment and boredom. For self-reported frustration, the variance explained by the model was moderate to high. For self-monitoring, the variance explained was moderate for sections 3 and 4 and low in section 2. Predictive relevance was largely moderate for self-reported frustration, except for T3, for which high predictive relevance was present. For self-monitoring, predictive relevance was low in sections 2 to 4, whereas, for sections 1 and 5, no predictive relevance emerged.

### Emotions and Self-Monitoring Predicting Mental Model Development

The bivariate Pearson correlations between aggregated values of self-reported emotions and self-monitoring, as well as pre- and post-test MMA, are given in Table 6. No significant correlation was found between self-reported enjoyment and self-monitoring at the aggregate level. Moreover, enjoyment appeared to be uncorrelated with pre- and post-test MMA. Self-reported boredom was significantly and negatively related to pre- as well as post-test MMA, but unrelated to self-monitoring. Conversely, self-reported frustration was related significantly and negatively to self-monitoring, but no significant correlation was found with either pre- or post-test MMA. Finally, a significant positive correlation was found between self-monitoring and post-test MMA.

**Table 6:** Pearson correlations between aggregated learner-state variables and pre- and post-test MMA.

Variable	1	2	3	4	5
1. Enjoyment	-				
2. Boredom	-0.19^*^	-			
3. Frustration	-0.45^***^	0.27^**^	-		
4. Self-Monitoring	0.09	-0.14	-0.23^*^	-	
5. Pre-test MMA	0.18	-0.21^*^	-0.01	0.13	-
6. Post-test MMA	0.05	-0.27^**^	-0.02	0.23^*^	0.50^***^


*MMA, mental model accuracy. *n* = 108. *^∗^p* < 0.05*, ^∗∗^p* < 0.01*, ^∗∗∗^p* < 0.001.*

The positioning of the aggregated variables in the subsequent PLS-PM analysis (see Figure 7) was made in accordance with the findings from the panel model analyses (see “Interrelations Between Emotions and Self-Monitoring” section). Since the relations between each emotion and self-monitoring in the panel models were unidirectional (i.e., no significant reciprocal relations emerged), the interrelations in the aggregate PLS-PM could be modeled unambiguously. Thus, we modeled self-reported enjoyment to be predicted by self-monitoring, whereas self-reported boredom and frustration were included as predictors of self-monitoring. Moreover, we modeled all three emotions, self-monitoring and pre-test MMA as predictors of post-test MMA. Finally, pre-test MMA was modeled as a predictor of emotions and self-monitoring (see Figure 7). For all predictors, the VIF ranged from 1.08 to 1.39 and the tolerance values ranged from 0.72 to 0.93. Thus, collinearity was not considered critical to the analysis (see Hair et al., 2013).

The results shown in Figure 7 illustrate that pre-test MMA was the strongest predictor for learners’ MMA after playing *Cure Runners*, showing a positive effect (see also Supplementary Table S4). Additionally, pre-test MMA was a significant negative predictor for self-reported boredom, indicating that learners with higher initial MMA experienced less boredom while playing *Cure Runners*. Furthermore, the amount of enjoyment reported while playing *Cure Runners* did not significantly predict post-test MMA. In contrast, self-reported boredom emerged as an additional significant and negative predictor of post-test MMA beyond pre-test MMA, but was unrelated to self-monitoring. Conversely, self-reported frustration while playing *Cure Runners* had no direct path to post-test MMA. Instead, frustration was a significant and negative predictor for self-monitoring. In addition, self-monitoring emerged as a significant predictor of MMA after playing *Cure Runners*, showing a positive effect. Thus, the more that learners engaged themselves in self-monitoring behavior while playing *Cure Runners*, the more accurate their mental models were afterward. However, there was no significant path between self-monitoring and enjoyment at the aggregate level. Concerning post-test MMA, the predictors explained a large amount of variance, whereas, for self-reported emotions and self-monitoring, the explained variance was low. In addition, the *Q*^2^ values indicate moderate predictive relevance for the post-test MMA and low predictive relevance for self-reported boredom and self-monitoring. With *Q*^2^ values close to and below 0, no predictive relevance was present for self-reported enjoyment and frustration.

## Discussion

Although multimedia learning environments are deemed to facilitate the development of accurate mental models (Moreno and Mayer, 2007; Sitzmann, 2011), affective and metacognitive learner-state variables can influence the effectiveness of multimedia learning (Moreno, 2005). In this study, we aimed to gain a deeper understanding of how learning-centered emotions and metacognitive self-monitoring interrelate and predict mental model development in multimedia learning. To this end, we collected repeated self-reports of three discrete emotions (i.e., enjoyment, boredom and frustration) while learners played a serious game as a specific form of a multimedia learning environment. In addition, a behavioral indicator was used to measure self-monitoring while learning. Thus, it was possible to account for the temporal dynamics between both learner-state variables. The presence of dynamic changes in emotions and self-monitoring was indicated by the preliminary analyses, where significant differences were found for the two learner-state variables over the course of the learning episode. To capture mental model development, a structural knowledge assessment was applied to assess the accuracy of learners’ knowledge, as organized in mental models (i.e., MMA) before and after the learning episode. Two main research questions were addressed in two consecutive analytical steps, in both of which PLS-PM analyses were applied.

### Relations and Temporal Dynamics Between Learning-Centered Emotions and Self-Monitoring

Regarding the first research question about the nature of the relationship between emotions and self-monitoring during learning, three major findings emerged. Firstly, self-reported enjoyment largely showed a positive relation to self-monitoring, whereas self-reported boredom and frustration were both negatively related to self-monitoring. This conforms to our hypothesis as well as to previous findings on the relations of positive and negative emotions with metacognitive strategy use (e.g., Perry et al., 2001; Pekrun et al., 2002, 2011; Mega et al., 2014). On a general note, the relations between emotions and self-monitoring appeared to be strongest for frustration. This can be derived from the higher number of significant correlations between self-monitoring and frustration as compared to enjoyment and boredom. In addition, self-reported frustration shared the highest number of significant indirect paths with self-monitoring in the panel models. Finally, frustration was the only emotion showing a significant correlation with self-monitoring when values were aggregated across the multimedia learning episode. The finding regarding the relative strengths of relations is not consistent with results from previous studies, based on aggregated measures of emotions as well as of metacognitive strategies (e.g., Mega et al., 2014; Pekrun et al., 2017). This discrepancy may be partly attributed to a bias, originating from retrospective emotion self-reports which are detached from a specific learning episode (see Mega et al., 2014). In particular, there exists evidence that positive learning-centered emotions, such as enjoyment, are more salient for learners than negative learning-centered emotions (Raccanello et al., 2018). Thus, retrospective self-reports of positive emotions may be more reliable than of negative emotions, which can also contort their relations with other variables. In addition, previous studies did not account for the dynamics of emotions during a learning episode (see Pekrun et al., 2017). Therefore, possible differences in the associations between emotions and self-monitoring which arise from differences in the intensities of emotional experience (see D’Mello and Graesser, 2014) could not be detected. Accordingly, it can be argued that boredom, in contrast with frustration, was not experienced with sufficient intensity in the present study in order to demonstrate a higher relation with self-monitoring. This view is further supported by the relatively low values of boredom, compared to frustration, as reported by learners during the learning episode.

The second major finding concerns the temporal dynamics between learning-centered emotions and self-monitoring, which differed for the three emotions. While self-reported enjoyment was elevated in consequence of heightened self-monitoring, opposite successions emerged for the negative emotions. Specifically, higher degrees of self-reported boredom and frustration were both followed by a decrease in self-monitoring in subsequent sections of *Cure Runners*. Thus, the prevalent assumption of emotions generally predicting metacognitive strategy use (e.g., Mega et al., 2014) was not completely supported by our results. However, the result conforms to the assumption that engaging in self-regulation strategies, such as self-monitoring, may lead to greater subjective control and, hence, greater enjoyment of a learning episode (e.g., Pekrun et al., 2002). In the present study, a high amount of self-monitoring behavior can be seen as engagement in self-regulation. Conversely, a low amount of self-monitoring may not have denoted increased perceived external control, given that learners could act freely within *Cure Runners*. Therefore, it is plausible that self-monitoring only preceded enjoyment, whereas it was not predictive of frustration and boredom. Instead, the negative emotions apparently arose from other stimuli (e.g., game events) during the learning episode (see Gilleade et al., 2005; van Lankveld et al., 2010; D’Mello, 2013) and subsequently predicted self-monitoring.

Thirdly, the relations between emotions and self-monitoring did not appear consistently throughout the learning episode. Most interestingly, self-reported boredom and frustration did not always have an immediate effect but showed indirect effects, predicting self-monitoring at later instances of the learning episode. This implies that boredom and frustration may need to persist over time in order to have an effect on learners’ self-monitoring. This is partly in line with assumptions that relate prolonged frustration to disengagement from learning (D’Mello and Graesser, 2012).

### Learning-Centered Emotions and Self-Monitoring as Predictors of Mental Model Development

The second research question addressed the effects of both learner-state variables on the accuracy of learners’ mental models after the multimedia learning episode. These effects were tested using aggregated values of learning-centered emotions and self-monitoring, with initial MMA (i.e., pre-test MMA) being controlled for. Initial MMA emerged as the strongest predictor of MMA after learning and also negatively predicted self-reported boredom averaged across all measurement occasions. This parallels findings which have related higher prior knowledge to less boredom experienced in learning activities (Pekrun et al., 2014; Putwain et al., 2017) and to more engaged concentration in multimedia learning environments (Shute et al., 2015). In line with Pekrun (2006), learners with relatively low initial MMA may have perceived less control over the learning episode, leading to a heightened experience of boredom.

Self-reported enjoyment was modeled to predict post-test MMA and self-monitoring, according to the temporal dynamics that emerged from the panel model analyses. Nevertheless, contrary to our hypothesis, enjoyment showed no significant relation to any of the two variables. Consistent with the low number of significant paths found between enjoyment and self-monitoring in the panel model, this result backs the notion that the relation was generally weak in the present study (see “Relations and Temporal Dynamics Between Learning-Centered Emotions and Self-Monitoring” section). Considering previous results regarding the relationship between positive emotions and mental model development (Bless, 2000), the present finding seems contradictory. However, Linnenbrink and Pintrich (2004) similarly find no evidence of an effect of positive emotions on mental model development. In addition, previous studies found that enjoyment experienced during single sessions of multimedia learning did not influence learning outcomes (Forsyth et al., 2015; Iten and Petko, 2016). Yet, it could still be that enjoyment experienced in multimedia learning environments affects mental model development “in the long run,” by stimulating learners to repeatedly engage in the learning episode (Garris et al., 2002).

The negative effect of self-reported boredom on post-test MMA, beyond the effect of pre-test MMA, is in line with our hypothesis and with previous research (e.g., Craig et al., 2004; Pekrun et al., 2014; Sabourin and Lester, 2014; Putwain et al., 2017). However, the path between boredom and self-monitoring was not significant in the aggregate model. Therefore, self-reported boredom may have predicted post-test MMA more strongly via other factors, such as low intrinsic motivation (see Pekrun et al., 2010). Moreover, boredom may have affected qualitative rather than quantitative aspects of self-monitoring, such as by reducing the quality of concentration (Hamilton et al., 1984). It has to be noted that boredom exerted its negative effect on mental model development, despite being experienced at relatively low levels in the present study (see “Relations and Temporal Dynamics Between Learning-Centered Emotions and Self-Monitoring” section). Thus, the present finding highlights the particularly grave effect of boredom on multimedia learning (see Baker et al., 2010).

In contrast to boredom, self-reported frustration during the learning episode also predicted self-monitoring at the aggregate level. Yet, unlike boredom and contrary to our hypothesis, frustration did not directly predict post-test MMA. It is possible that frustration had an additional positive effect which compensated for the lack of self-monitoring as an effective way to increase MMA. Thus, frustrated learners may have disengaged from self-monitoring and instead invested more effort into other beneficial processes or strategies, such as critical thinking or elaboration (see Pekrun et al., 2017). Evidence of such a beneficial role played by frustration in multimedia learning comes from Shute et al. (2015), who showed that frustration led to higher in-game performance in a physics game. However, similar to the present study, there was no significant effect of frustration on learning outcomes (Shute et al., 2015). Together with the present findings, this may point to a twofold role of frustration in multimedia learning, with detrimental and beneficial effects partly canceling each other out.

Finally, self-monitoring emerged as a significant positive predictor for post-test MMA over and above the effect of pre-test MMA, which conforms to our hypothesis. In addition, this finding provides further support for the crucial role of self-monitoring for mental model development in multimedia learning (see also Greene and Azevedo, 2009; Roscoe et al., 2013; Riemer and Schrader, 2016a). In the context of the present study, learners who spent more time viewing information about their progress may have successfully detected and resolved cognitive conflicts which emerged during the learning episode. Consequently, they achieved a deeper understanding about the relations between domain concepts of practical money skills, resulting in more accurate mental models after the learning episode.

### Limitations

The present study has several limitations. First, a limitation can be seen in the absence of a significant increase in MMA, for which there are several possible reasons. In relation to the results of this study, one reason may be that learners were generally engaged in low degrees of self-monitoring as an important predictor for post-test MMA. More specifically, learners spent only an average of 5% of the time while on the decision and reflection phases viewing self-monitoring. Likewise, in previous studies on multimedia learning, authors also noted that learners might have been insufficiently engaged in self-monitoring and, thus, failed to show an increase in MMA (Roscoe et al., 2013; Riemer and Schrader, 2016a). A further reason for the non-significant increase in MMA may be that learners were not sufficiently motivated to learn because the objective of *Cure Runners* was not part of the institution’s curriculum. However, we argue that the topic (i.e., practical money skills) was of relevance to the learners since university students usually have limited time resources to generate income and, thus, rely on shrewd money management (see also Gerrans and Heaney, 2016). Nevertheless, future studies should aim to investigate the role of learner-state variables in multimedia learning environments when embedded into a course curriculum in order to replicate the results found in the present study.

A second limitation concerns the selection and measurement of the emotions experienced during the learning episode. Enjoyment, boredom and frustration have been chosen because of their relevance as activity-related learning-centered emotions (see Pekrun, 2006). However, additional epistemic emotions and affective-cognitive states, such as curiosity or confusion (e.g., D’Mello and Graesser, 2012; Pekrun et al., 2017), will need to be considered in future studies. Furthermore, the repeated collection of self-reports of emotions can be criticized, since this could have interrupted the learning experience (Pagulayan et al., 2003). Moreover, repeated self-reports of emotions may distort the effects of emotions on cognitive and metacognitive processes (Spering et al., 2005). Therefore, future studies should seek to apply objective and less invasive measures of emotions, for example, by using input devices (e.g., Miller and Mandryk, 2016) or recording facial or bodily expression features (e.g., Wiklund et al., 2015; Riemer et al., 2017).

Thirdly, the measure of self-monitoring was purely quantitative, in that only a weighted measure of time spent on specific game screens was used. Therefore, it did not provide information about how this time was actually being used by learners. As indicated above, this may have been a reason for the relatively weak relation found between self-monitoring and self-reported boredom. Although this measure provided non-invasive insights into a relevant learner-state variable, future studies should seek to identify assessments of in-game self-monitoring which are more refined, for example, by capturing self-monitoring accuracy (see de Bruin and van Gog, 2012).

Finally, the present research design was chosen over an experimental manipulation for the benefit of a comprehensive investigation into the temporal dynamics between the three discrete emotions and self-monitoring in a naturalistic setting. Consequently, the results only provide limited support for causal effects (see Selig and Little, 2012). Moreover, the interrelations between emotions and self-monitoring over time were investigated in three separate panel models, in order to achieve converging and interpretable path models. However, no effects of interrelated emotions (i.e., mixed emotions) could be detected with this approach. In addition, the aggregation of variables in the second step of our analytical approach may have yielded a decrease in statistical power (see Bakdash and Marusich, 2017). Nevertheless, the present findings point to effects that go beyond mere associational relations, given the temporal precedence of the variables in the panel models. Thus, this study may aid future experimental research focusing on causal effects between learning-centered emotions and metacognitive self-monitoring.

## Conclusion

The present study highlights the crucial role of learning-centered emotions in multimedia learning. In particular, negative emotions, such as boredom and frustration, appear to hinder cognitive and metacognitive processes relevant for mental model development. In addition, the findings stress the importance of engaging in metacognitive self-monitoring during multimedia learning in order to develop accurate mental models. Moreover, it could be shown that positive and negative emotions interrelate with self-monitoring in different ways. The findings from this study can aid researchers in making more accurate predictions about the effects of learning-centered emotions in future studies. As for practical implications, findings from the present study can support the development of more efficient multimedia learning environments which can adapt to learners’ states. In particular, our results imply that negative emotions, such as frustration, may not need to be reduced as soon as they are detected (e.g., by reducing the task difficulty). Conversely, frustration should neither go unattended, given the negative impact it may have during the later stages of the learning episode. Moreover, the lack of a direct impact of frustration on mental model development leaves room for possible compensating effects to be investigated. In refining the measurement of emotions during multimedia learning, it may be possible to detect the thresholds for detrimental and beneficial levels of frustration.

## Ethics Statement

This study was carried out in accordance with the recommendations of the ethical committee of Ulm University with written informed consent from all subjects. All subjects gave written informed consent in accordance with the Declaration of Helsinki. The ethics committee of Ulm University exempted the study from the full ethics review process and declared that an ethics approval was not required for this study as per applicable guidelines and national regulations.

## Author Contributions

All authors contributed to the conception of the work. VR designed and conducted the study and conducted the statistical analyses. VR and CS wrote the first draft of the manuscript. All authors revised the final manuscript.

## Conflict of Interest Statement

The authors declare that the research was conducted in the absence of any commercial or financial relationships that could be construed as a potential conflict of interest.
