# Corrigendum: Mental Model Development in Multimedia Learning: Interrelated Effects of Emotions and Self-Monitoring

**DOI:** 10.3389/fpsyg.2019.01174

**Published:** 2019-05-22

**Authors:** Valentin Riemer, Claudia Schrader

**Affiliations:** Institute of Psychology and Education, Ulm University, Ulm, Germany

**Keywords:** interactive learning environments, serious games, mental models, metacognition, emotion

In the original article, the terms “Section” and “Sections” have been used to denote the five consecutive game sections of the stimulus game *Cure Runners*. This led to a confusion in the production process of the manuscript, where the respective numbers of the (game) sections have been mistakenly replaced by the headings of the article sections.

The following corrections have been made:

In the **Results**, subsection **Descriptive Statistics and Preliminary Analyses**, paragraph two:

“Self-monitoring appeared to be exhibited to a higher degree during the three middle sections compared to the first and last sections (see Table 1). There were significant differences in self-monitoring between sections [*F*_(4, 84)_ = 41.63, *p* < 0.001, $\eta_{p}^{2}$ = 0.67]. In addition, the amounts of self-monitoring differed significantly between almost all sections of *Cure Runners*, except between sections 2 and 3 as well as between sections 2 and 4 (see Table 1).”

In the **Results**, subsection **Enjoyment**, paragraph one and two:

“The bivariate Pearson correlations between the single measurement occasions of self-reported enjoyment and self-monitoring, shown in Table 3, were largely positive. Thus, learners who reported higher enjoyment while playing *Cure Runners* also engaged in more self-monitoring. However, only the correlation coefficients between enjoyment at T2 and T4 and self-monitoring in section 4 were statistically significant.”

“The results of the PLS-PM analysis are presented in Figure 4 (see also Table S1 in the Supplementary Materials). No significant paths from self-reported enjoyment in the direction of self-monitoring were found over the course of playing *Cure Runners*. In contrast, self-monitoring, as exhibited during section 2, was a significant positive predictor of enjoyment reported at T2 (i.e., after section 2). Additionally, there were significant positive indirect paths from self-monitoring to self-reported enjoyment. The indirect paths shown in Figure 4 represent the compound effects of all direct paths between self-monitoring in section 2 and enjoyment reported at T3, T4, and T5. Furthermore, enjoyment and, to a lesser extent, self-monitoring showed significant autoregressive effects. The *R*^2^ values presented in Figure 4 indicate that the variance explained by the predictors was moderate to high for self-reported enjoyment and small to moderate for self-monitoring. In addition, the *Q*^2^ values indicate that predictive relevance in the model was medium to high for all five measurement occasions of enjoyment and low for self-monitoring in sections 2 to 4. For self-monitoring in sections 1 and 5, predictive relevance was not given.”

In the **Results**, subsection **Boredom**, paragraph two:

“The PLS-PM presented in Figure 5 (see also Table S2 in the Supplementary Materials) shows that boredom reported at T3 was a significant negative predictor of self-monitoring exhibited in the subsequent section (4). In addition, significant indirect paths were found for boredom reported at T1 and T2, negatively predicting self-monitoring in section 4. No significant paths of self-monitoring on the subsequent reports of boredom were found. Moreover, the autoregressive effects of boredom and self-monitoring were similar to those in the model with self-reported enjoyment and self-monitoring. The explained variance was high for self-reported boredom and low for self-monitoring, except for section 4 where the *R*^2^ value was of a moderate size. Predictive relevance was moderate to high for self-reported boredom and low for self-monitoring, except in sections 1 and 5, in which no predictive relevance was present.”

In the **Results**, subsection **Frustration**, paragraph one and two:

“The Pearson correlations for the measurement occasions of self-reported frustration and self-monitoring are provided in Table 5, revealing largely negative correlations between frustration and self-monitoring. Thus, learners who reported more frustration also showed less self-monitoring. However, significant correlation coefficients were only found between the final four measurement occasions of frustration and self-monitoring during sections 3 and 4.”

“The results of the PLS-PM analysis, presented in Figure 6, indicate that frustration reported at T2 was a significant negative predictor of self-monitoring during the subsequent section (see also Table S3 in the Supplementary Materials). Additionally, a series of significant indirect paths was detected. The dashed lines in Figure 6 represent the compound effects of all direct paths between frustration reported at the baseline, at T1 and at T2, in turn negatively predicting self-monitoring in sections 3 and 4. No significant paths were found for self-monitoring in the direction of self-reported frustration. The autoregressive effects in the model resemble those reported for the models with self-reported enjoyment and boredom. For self-reported frustration, the variance explained by the model was moderate to high. For self-monitoring, the variance explained was moderate for sections 3 and 4 and low in section 2. Predictive relevance was largely moderate for self-reported frustration, except for T3, for which high predictive relevance was present. For self-monitoring, predictive relevance was low in sections 2 to 4, whereas, for sections 1 and 5, no predictive relevance emerged.”

The authors apologize for these errors and state that they do not change the scientific conclusions of the article in any way. The original article has been updated.

