# Supplementary material for: Mental Model Development in Multimedia Learning: Interrelated Effects of Emotions and Self-Monitoring
Source: Front Psychol. 2019 Apr 24;10:899. doi: 10.3389/fpsyg.2019.00899 (PMC6491813; doi:10.3389/fpsyg.2019.00899)
Supplement: Supplementary file 1 [file Table_1.docx]

Supplementary Material

Table S1 Beta coefficients with bootstrap confidence intervals for paths between single measurement occasions of enjoyment and self-monitoring

| Autoregressive paths | β | 95 % Bootstrap CI | Cross paths | β | 95 % Bootstrap CI |
| --- | --- | --- | --- | --- | --- |
| En BL → En T1 | .55*** | [.412; .691] | En BL → S-m S1 | -.03 | [-.252; .203] |
| En T1 → En T2 | .51*** | [.324; .663] | En T1 → S-m S2 | -.16 | [-.385; .075] |
| En T2 → En T3 | .66*** | [.482; .801] | En T2 → S-m S3 | .15 | [-.011; .304] |
| En T3 → En T4 | .67*** | [.516; .789] | En T3 → S-m S4 | .13 | [-.104; .345] |
| En T4 → En T5 | .48*** | [.255; .668] | En T4 → S-m S5 | .15 | [-.066; .368] |
| S-m S1 → S-m S2 | .29** | [.115; .467] | S-m S1 → En T1 | .07 | [-.121; .264] |
| S-m S2 → S-m S3 | .22* | [.033; .393] | S-m S2 → En T2 | .22* | [.045; .373] |
| S-m S3 → S-m S4 | .28** | [.091; .510] | S-m S3 → En T3 | .08 | [-.088; .243] |
| S-m S4 → S-m S5 | -.01 | [-.227; .236] | S-m S4 → En T4 | .11 | [-.010; .243] |
|  |  |  | S-m S5 → En T5 | -.15 | [-.335; .037] |

*Note.* En = enjoyment. S-m = self-monitoring. BL = baseline measurement. T1 to T5 = five measurement occasions during playing *Cure Runners*. S1 to S5 = five sections of *Cure Runners*. *n* = 88*. * p* < .05*. ** p* < .01*. *** p* < *.*001.
