# Supplementary material for: Mental Model Development in Multimedia Learning: Interrelated Effects of Emotions and Self-Monitoring
Source: Front Psychol. 2019 Apr 24;10:899. doi: 10.3389/fpsyg.2019.00899 (PMC6491813; doi:10.3389/fpsyg.2019.00899)
Supplement: Supplementary file 2 [file Table_2.docx]

Supplementary Material

Table S2 Beta coefficients with bootstrap confidence intervals for paths between single measurement occasions of boredom and self-monitoring

| Autoregressive paths | β | 95 % Bootstrap CI | Cross paths | β | 95 % Bootstrap CI |
| --- | --- | --- | --- | --- | --- |
| Bo BL → Bo T1 | .57*** | [.380; .725] | Bo BL → S-m S1 | .06 | [-.172; .299] |
| Bo T1 → Bo T2 | .66*** | [.481; .809] | Bo T1 → S-m S2 | -.01 | [-.250; .225] |
| Bo T2 → Bo T3 | .65*** | [.426; .839] | Bo T2 → S-m S3 | -.07 | [-.276; .150] |
| Bo T3 → Bo T4 | .79*** | [.656; .888] | Bo T3 → S-m S4 | -.19* | [-.328; -.031] |
| Bo T4 → Bo T5 | .72*** | [.537; .850] | Bo T4 → S-m S5 | .13 | [-.023; .301] |
| S-m S1 → S-m S2 | .28** | [.102; .451] | S-m S1 → Bo T1 | -.09 | [-.236; .065] |
| S-m S2 → S-m S3 | .23* | [.043; .416] | S-m S2 → Bo T2 | -.11 | [-.293; .063] |
| S-m S3 → S-m S4 | .27** | [.099; .494] | S-m S3 → Bo T3 | -.11 | [-.227; .009] |
| S-m S4 → S-m S5 | .04 | [-.165; .267] | S-m S4 → Bo T4 | .10 | [-.027; .262] |
|  |  |  | S-m S5 → Bo T5 | -.04 | [-.185; .141] |

*Note.* Bo = boredom. S-m = self-monitoring. BL = baseline measurement. T1 to T5 = five measurement occasions during playing *Cure Runners*. S1 to S5 = five sections of *Cure Runners*. *n* = 88*. * p* < .05*. ** p* < .01*. *** p* < *.*001.
