# Supplementary material for: Mental Model Development in Multimedia Learning: Interrelated Effects of Emotions and Self-Monitoring
Source: Front Psychol. 2019 Apr 24;10:899. doi: 10.3389/fpsyg.2019.00899 (PMC6491813; doi:10.3389/fpsyg.2019.00899)
Supplement: Supplementary file 3 [file Table_3.docx]

Supplementary Material

Table S3 Beta coefficients with bootstrap confidence intervals for paths between single measurement occasions of frustration and self-monitoring

| Autoregressive paths | β | 95 % Bootstrap CI | Cross paths | β | 95 % Bootstrap CI |
| --- | --- | --- | --- | --- | --- |
| Fr BL → Fr T1 | .45*** | [.272; .611] | Fr BL → S-m S1 | -.02 | [-.271; .267] |
| Fr T1 → Fr T2 | .49*** | [.311; .653] | Fr T1 → S-m S2 | .02 | [-.189; .227] |
| Fr T2 → Fr T3 | .60*** | [.439; .740] | Fr T2 → S-m S3 | -.28*** | [-.430; -.131] |
| Fr T3 → Fr T4 | .55*** | [.358; .712] | Fr T3 → S-m S4 | -.19 | [-.394; .014] |
| Fr T4 → Fr T5 | .55*** | [.369; .713] | Fr T4 → S-m S5 | .05 | [-.184; .279] |
| S-m S1 → S-m S2 | .28** | [.098; .462] | S-m S1 → Fr T1 | -.15 | [-.325; .024] |
| S-m S2 → S-m S3 | .23* | [.059; .391] | S-m S2 → Fr T2 | -.02 | [-.201; .161] |
| S-m S3 → S-m S4 | .26* | [.067; .481] | S-m S3 → Fr T3 | -.10 | [-.245; .067] |
| S-m S4 → S-m S5 | .04 | [-.178; .272] | S-m S4 → Fr T4 | -.10 | [-.282; .061] |
|  |  |  | S-m S5 → Fr T5 | .06 | [-.092; .218] |

*Note.* Fr = frustration. S-m = self-monitoring. BL = baseline measurement. T1 to T5 = five measurement occasions during playing *Cure Runners*. S1 to S5 = five sections of *Cure Runners*. *n* = 88*. * p* < .05*. ** p* < .01*. *** p* < *.*001.
