# Supplementary material for: Mental Model Development in Multimedia Learning: Interrelated Effects of Emotions and Self-Monitoring
Source: Front Psychol. 2019 Apr 24;10:899. doi: 10.3389/fpsyg.2019.00899 (PMC6491813; doi:10.3389/fpsyg.2019.00899)
Supplement: Supplementary file 4 [file Table_4.docx]

Supplementary Material

Table S4 Beta coefficients with bootstrap confidence intervals for paths between aggregated learner-state variables, pre-, and post-test MMA

| Paths | β | 95 % Bootstrap CI |
| --- | --- | --- |
| Pre-test MMA → Post-test MMA | .45*** | [.267; .612] |
| Pre-test MMA → Enjoyment | .17 | [-.011; .345] |
| Pre-test MMA → Boredom | -.21* | [-.384; -.038] |
| Pre-test MMA → Frustration | -.01 | [-.205; .172] |
| Pre-test MMA → Self-monitoring | .11 | [-.077; .295] |
| Enjoyment → Post-test MMA | -.05 | [-.215; .108] |
| Boredom → Post-test MMA | -.17* | [-.326; -.021] |
| Boredom → Self-monitoring | -.05 | [-.243; .134] |
| Frustration → Post-test MMA | .04 | [-.135; .225] |
| Frustration → Self-monitoring | -.21* | [-.374; -.030] |
| Self-monitoring → Post-test MMA | .17* | [.005; .323] |
| Self-monitoring → Enjoyment | .07 | [-.118; .235] |

*Note.* MMA = mental model accuracy. *n* = 108*. * p* < .05*. ** p* < .01*. *** p* < *.*001.
